# Why is it important? Financial literacy in students in entrepreneurship: A systematic literature review

**DOI:** 10.12688/f1000research.160829.1

**Published:** 2025-01-27

**Authors:** Dwi Nanda Akhmad Romadhon, Hari Mulyadi

**Affiliations:** 1 Universitas Pendidikan Indonesia, West Java, Indonesia; 2 Universitas Pendidikan Indonesia, West Java, Indonesia

**Keywords:** Financial Literacy, Entrepreneurship, Systematic Literature Review

## Abstract

This paper aims to analyze financial literacy in students a foundational understanding of personal finance management in addition to more intricate financial concepts like corporate risk analysis and capital management techniques. A comprehensive evaluation of the literature has been conducted by locating 155 studies from different sources. 36 papers were determined to be pertinent for the current investigation after the 155 research were eliminated based on selection criteria. The results of the study show that financial literacy has a significant impact on the success of students in entrepreneurship, both in planning, management, and business development. This article also provides recommendations for the development of a more comprehensive entrepreneurship education curriculum, which integrates financial literacy as a core competency. This paper develops a research model that can be used for universities or policymakers who need to create financial skills in entrepreneurial students in order to improve financial performance.

## Introduction

Due to the increasing emphasis on entrepreneurship education and supportive university initiatives, entrepreneurship is increasingly recognized as a viable career choice among students worldwide. Studies show that universities are actively strengthening their support systems to encourage students to be entrepreneurs, especially those who are somewhat proactive ( Mustafa et al., 2023). In today’s era of globalization and digitalization, opportunities to start and develop businesses are increasingly wide open, especially for the younger generation who have access to technology and information ( Kumar, 2024). According to research, the entrepreneurship landscape has become easier for young people, mainly because they have access to technology and data in the digital age. Digital platforms such as e-commerce and social media allow young people to create innovations and transform conventional industries.

However, success in the business world is not only determined by innovation and creativity alone, but also by the ability to manage finances effectively. Financial literacy, which includes an understanding of personal financial management, financial planning, investing, and risk management, is an important factor that can influence an entrepreneur’s success. Businesses, including MSMEs, are successful because financial skills help them make informed financial decisions, identify opportunities, and ensure long-term growth and sustainability ( Dwyanti, 2024). The problem of low financial literacy among students has been studied in a variety of contexts, revealing common problems and unique insights. However, many students around the world have low financial literacy, which can have significant consequences ( Abdul Karim et al., 2023).

Unfortunately, various studies show that the level of financial literacy among college students, including those interested in entrepreneurship, is still relatively low. Despite the acknowledged significance, research shows that the level of financial intelligence of college students including those interested in entrepreneurship remains very low. Many studies draw attention to various aspects of this issue, because financial skills support personal wealth management and business decision-making, it is especially important for college students, especially those who intend to pursue entrepreneurship ( Li, 2024).

Ignorance or lack of understanding of the basic principles of finance can negatively impact students’ ability to make informed financial decisions, manage cash flow, and avoid risks that could lead to business failure. In this context, financial literacy is not just an additional skill, but an essential element that can determine the success or failure of a business ( Rehman & Mia, 2024). The importance of financial skills in influencing individual and organizational financial decision-making is increasingly recognized as a key factor in determining company performance.

**Figure 1.**
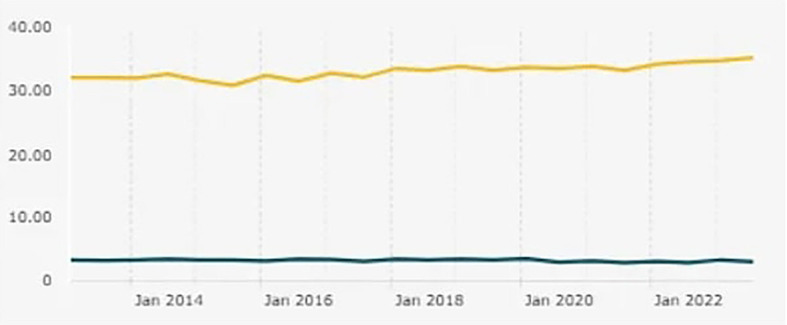
Entrepreneurship Ratio in Indonesia. Note: This figure/table has been reproduced with permission from https://databoks.katadata.co.id/ketenagakerjaan/statistik/db2da5b8fde8716/ini-perkembangan-rasio-wirausaha-indonesia-sampai-2023.

The ratio of entrepreneurs in Indonesia from year to year has not experienced the expected increase ( Katadata, 2023). Figure 1 illustrates the entrepreneurship ratio in Indonesia, highlighting its comparison with the minimum standard in developed countries. According to Teten Masduki, Minister of Cooperatives and SMEs (MenKopUKM), the number of entrepreneurs in Indonesia is very large, reaching 64 million people, but the ratio of new entrepreneurs is only 3.47 percent. He said that developed countries must have an entrepreneurship ratio of at least 4 percent. Meanwhile, Arif Satria, Rector of Universitas IPB, said that 43% of new students aspire to become entrepreneurs. To realize the dreams of these students, IPB is preparing various programs to become an incubator that is able to give birth to new entrepreneurs in Indonesia ( Kemenkopukm, 2023).

In the world of entrepreneurship, financial skills play an important role, significantly influencing business performance, decision-making, and economic empowerment. For example, Srivastava’s study emphasizes that financial skills improve women’s confidence, decision-making abilities, and access to resources, thereby improving business performance and promoting entrepreneurial success ( Prabha, 2024).

In the current digital economy era, entrepreneurship is one of the career options that is increasingly in demand by students. However, success in entrepreneurship is not only determined by innovation and creativity, but also by the ability to manage finances effectively. Financial literacy, which includes an understanding of financial planning, risk management, and investing, is an important competency for students who want to enter the business world ( Sumantri, Alit & Indraswari, Paramita, Ayu, Agung, Gusti, 2024).

Seeing the importance of financial literacy in the world of entrepreneurship, this study aims to systematically review the existing literature related to the topic using the PRISMA (Preferred Reporting Items for Systematic Reviews and Meta-Analyses) method, this research will identify, filter, and analyze relevant research to understand the role of financial literacy in student entrepreneurship. This literature review is expected to provide deeper insights into the importance of financial literacy in entrepreneurship education, as well as provide recommendations for the development of a more comprehensive curriculum in higher education.

Thus, this research will make a significant contribution not only to academics and researchers, but also to education policymakers and entrepreneurial practitioners. The results of this literature review can be the basis for efforts to improve financial literacy among students, so that they are better prepared to face challenges in the business world and become successful entrepreneurs.

## Methods

### Research design

This study uses a systematic literature review method with PRISMA guidance. This method involves four main stages: identification, screening, feasibility, and inclusion ( Page et al., 2021).

### PRISMA procedure

**A. Identification**

**Figure 2.**
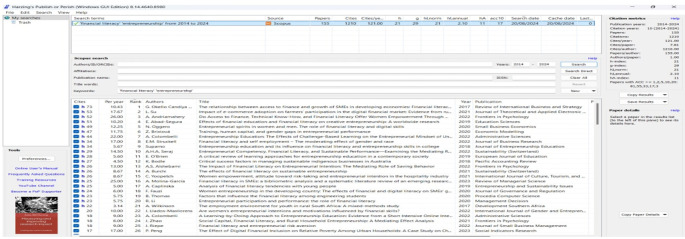
Scopus keyword search using publish or perish.

Relevant literature is identified through an academic database search from Scopus. Scopus with more than 22,794 titles and more than 78 million bibliographic records includes research journals, conference proceedings, and academic books from more than 5,000 publishers worldwide, and was selected as the main database for academic literature despite some limitations noted ( Gasparyan & Kitas, 2021). The keywords used include “financial literacy” and “entrepreneurship”. Source search using Publish or Perish software ( Harzing, 2024). The results of the Scopus keyword search, visualized using Publish or Perish, are shown in Figure 2.

Paper selection from 2014-2024 with an average number of citations of 10 per year. The number of papers obtained was 155 with a total of 1210 citations and an average of 121 citations, followed by 7.8 citations per paper. Scopus with more than 22,794 titles and more than 78 million bibliographic records includes research journals, conference proceedings, and academic books from more than 5,000 publishers worldwide, and was selected as the main database for academic literature despite some limitations noted ( Gasparyan & Kitas, 2021).

**B. Filtering**

Articles that are irrelevant, such as those that are not student-focused or entrepreneurial, are filtered by title and abstract. The study of the selection process is a process consisting of search, selection, and development, each of which is divided into a number of stages and performs different activities. To start your search, use the Publish or Perish electronic database, which has a Scopus output of 155 works of literature. In addition, the process consists of selecting articles that meet the criteria that have been set, as well as analyzing the development of the article.

**C. Eligibility**

Articles that pass the screening stage are further evaluated based on eligibility criteria, such as the research method used, the suitability of the topic, and the validity of the research results.

**D. Inclusion**

Articles that meet all eligibility criteria are included in the final review for further analysis. To ensure relevance and appropriateness in systematic reviews and other research methodologies, inclusion and exclusion criteria are an important component in selecting studies to conduct. They are used to determine which research is considered relevant and which is not, based on pre-defined parameters that are in accordance with the research objectives. Creating clear and clear criteria for inclusion and exclusion is critical to the decision-making process ( Tod, 2019).

**E. Inclusion and Exclusion Criteria**

Results are collected after the search is applied. Each study must be eligible for inclusion and removal. In the first section, 155 articles were reviewed based on inclusion and exclusion criteria. After that, the last 144 articles were selected for judging. This systematic literature review discusses financial literacy and entrepreneurship in students. The main questions discussed in this review are as follows: definition of financial literacy and ethics in students; Objectives, methodologies, and key outcomes of research on financial literacy and credit in students over the past 10 years.

• **Inclusion**: Research published in the last 10 years, using quantitative or qualitative methods, and focusing on financial literacy in the context of student entrepreneurship.
• **Exclusion**: Articles that are not in United Kingdom, are not peer-reviewed, or do not provide empirical data.

Inclusion research criteria will be incorporated into it, and research that does not meet them will be thoroughly reviewed to ensure that it meets certain quality standards. This overview focuses on quality standards. It concentrates on the description of the draft financial literacy in entrepreneurship, purpose research, design research, instruments, samples, answers to research questions, conclusions, weaknesses, and recommendations for financial literacy in entrepreneurship in college students. In the end, 36 articles were selected for analysis and used in research that answered the questions. Here are the total documents based on the type of documents sorted in Table 1.

**Table 1.** Sorting documents.

Document type	Sum
Article	115
Book	1
Book Chapters	16
Conference Papers	20
Editorials	1
Reviews	3

## Results and Discussion

### Result

**Figure 3.**
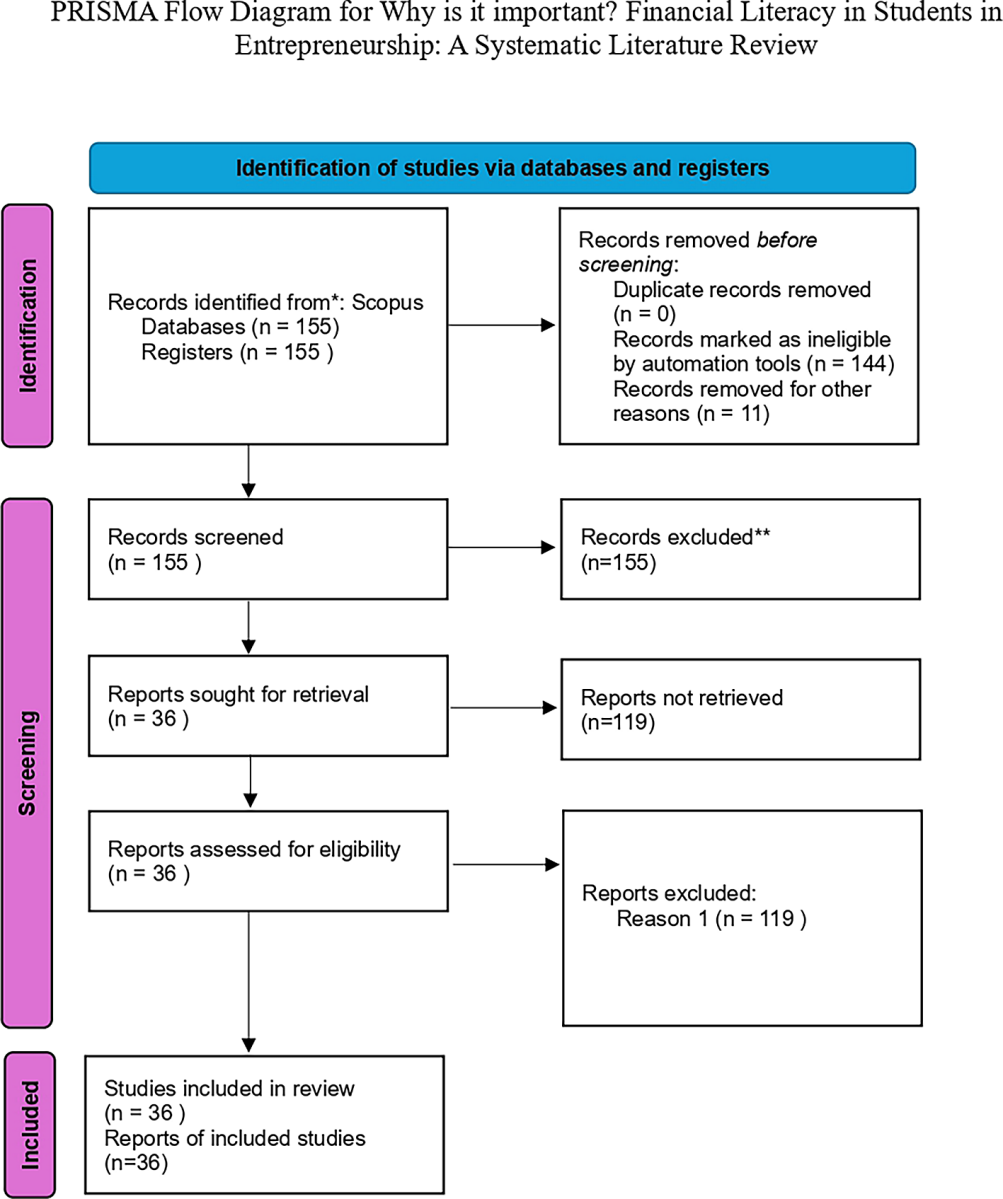
PRISMA flow diagram.

From the search results, a number of relevant articles were obtained as many as 115 papers, The distribution of citation counts across the analyzed articles is illustrated in Figure 3 ( Romadhon & Mulyadi, 2025).

### Discussion

The results of this literature review show that knowledge about finance should be part of the entrepreneurship education program at the university. There is also a need for more practical and relevant educational programs that teach students how to manage money.

It is essential to have a good understanding of finance, which should be thoroughly incorporated into the entrepreneurship education program in college. Students gain extensive financial knowledge. It not only teaches them the basics of personal financial management, such as budgeting, savings planning, investment, and debt management, but also helps them understand more complex financial dynamics, such as capital management strategies in business competition, and risk analysis when making investment decisions.

In addition, these results show that educational programs must be created on a theoretical and practical basis. Realistic case study analysis, business simulations, and involvement in real entrepreneurial projects should be part of a program like this. This method will allow students to use their understanding of finance in relevant and challenging situations. This will improve their abilities in financial analysis, business planning, and financial risk management.

A college entrepreneurship curriculum that integrates in-depth financial knowledge is expected to produce graduates who not only have a strong theoretical understanding, but are also practically prepared to face the complex financial challenges of the business world. Therefore, financial literacy can be a strategic tool that helps reduce the rate of business failure caused by a poor understanding of finance. In addition, financial literacy can prepare students to become smarter and more flexible business leaders in the face of constant economic changes.

Budgeting, cash flow monitoring, and investment allocation are just a few of the technical aspects of financial management, but financial literacy affects many things. A strong understanding of finance helps students gain more confidence when facing difficult and uncertain business challenges. With good financial knowledge, students can not only overcome various methods of capital management and financial instruments, but they can also make more careful and strategic decisions in a dynamic business environment. This includes looking for profitable investment opportunities, managing financial risk, and creating sustainable growth strategies.

In addition, entrepreneurship education that incorporates financial knowledge is also an important foundation for building a proactive and flexible entrepreneurial mentality. Students who have a good financial understanding tend to be better prepared to take calculated risks and more confident in the face of changing market pressures. They rely not only on basic knowledge or intuition, but also in-depth financial analysis and accurate calculations to help them make important decisions.

Therefore, the inclusion of financial knowledge into the high school entrepreneurship curriculum is very important. This step not only improves students’ technical skills in financial management, but also builds a resilient and confident mindset to manage a business in the real world. By understanding finance well, students will be better prepared to face the complex business world, better equipped to adapt to market changes, and ultimately will become successful and innovative entrepreneurs in the future.

## Conclusion

As part of the entrepreneurship education curriculum at universities, financial knowledge is essential, according to this literature review. Students must understand finance thoroughly. This includes basic knowledge of managing personal finances as well as more complex financial dynamics, such as capital management strategies and business risk analysis. Educational programs that combine theory with practice, such as case studies and business simulations, will help students apply their knowledge to real-world situations. Students who have financial knowledge will be more confident in facing business challenges, better able to make careful choices, and better equipped to become successful and flexible entrepreneurs as the economy changes.

Financial literacy is an essential competency that supports student success in entrepreneurship. Therefore, the integration of financial literacy in entrepreneurship education needs to be improved to produce young entrepreneurs who are competent and ready to face global economic challenges.

## Recommendations

• Development of a financial literacy module specific to entrepreneurship students.
• Increased collaboration between academics, business practitioners, and financial institutions to strengthen financial literacy education.

## Data Availability

No data are associated with this article. *Reporting guidelines* Zenodo: PRISMA Flow Diagram for Why is it important? Financial Literacy in Students in Entrepreneurship: A Systematic Literature Review [Data set]. Zenodo. https://doi.org/10.5281/zenodo.14639718 ( Romadhon & Mulyadi, 2025).

## Software availability statement

The software tools mentioned in the article are as follows:

**Publish or Perish:** This software is open-access and was used to retrieve bibliometric data for the PRISMA methodology. A hyperlink to the software has been added in the revised manuscript: https://harzing.com/resources/publish-or-perish/windows.

**Mendeley Citation Manager:** This software is a proprietary tool but is freely available for reference management purposes. To address your suggestion, we have provided an additional note in the Software Availability statement to indicate Mendeley’s availability and functionality. This has been acknowledged in the revised manuscript.

**Software availability:** The Publish or Perish software used for bibliometric analysis is freely available at https://harzing.com/resources/publish-or-perish/windows. Mendeley, a citation management tool used in this study, is freely available for download at https://www.mendeley.com.
