# Why is it important? Financial literacy in students in entrepreneurship: A systematic literature review

**DOI:** 10.12688/f1000research.160829.3

**Published:** 2025-10-31

**Authors:** Dwi Nanda Akhmad Romadhon, Hari Mulyadi

**Affiliations:** 1 Universitas Pendidikan Indonesia, West Java, Indonesia; 2 Universitas Pendidikan Indonesia, West Java, Indonesia

**Keywords:** Financial Literacy, Entrepreneurship, Systematic Literature Review

## Abstract

Student entrepreneurship is increasingly recognized as a driver of economic growth, innovation, and job creation, particularly in developing countries. However, limited financial literacy remains a major barrier to entrepreneurial success among students. This study aims to systematically review the role of financial literacy in supporting student entrepreneurship. A systematic search using the PRISMA 2020 method was conducted in Scopus for the period 2014–2024 with the following search string: (“financial literacy” AND entrepreneurship AND student*) OR (“financial literacy” AND “youth entrepreneurship”). From an initial 155 records, 36 studies met the inclusion criteria: peer-reviewed, empirical, English language, and focusing on students in the context of entrepreneurship. The review identifies three main themes. First, the financial skills addressed include budgeting, saving and borrowing, cash-flow management, investment and risk assessment, and basic accounting. Second, approaches to improving financial literacy are primarily through integrated curricula, business simulations, student venture projects, and industry mentoring. Third, financial literacy strongly impacts entrepreneurial outcomes, including higher self-efficacy, better financial decision-making, stronger resilience in cash-flow management, and greater sustainability of student ventures. The findings underscore the importance of integrating financial literacy as core human capital within entrepreneurship education, especially in developing countries. Limitations of this review include reliance on Scopus and exclusion of non-English studies. The study provides theoretical, practical, and policy implications for universities, educators, and policymakers.

## Introduction

Entrepreneurship is widely recognized as a driver of economic growth, job creation, and social innovation, and universities increasingly position entrepreneurship education and support programs as pathways for students to pursue entrepreneurial careers ( Mustafa et al., 2023). In the era of globalization and digitalization, the barriers to entry are lower for young people who can leverage data, e-commerce, and social media to innovate and transform conventional industries ( Kumar, 2024). Yet, innovation and creativity alone do not guarantee business success; the ability to manage finances effectively is equally decisive.

Financial literacy covering personal financial management, budgeting and cash-flow planning, saving and borrowing, risk assessment and investment, basic accounting, and capital management enables entrepreneurs to make informed decisions, identify opportunities, and ensure long-term sustainability ( Dwyanti, 2024). Evidence across contexts indicates that many university students still exhibit low levels of financial literacy, exposing them to costly mistakes, fraud, and fragile cash-flow positions ( Abdul Karim et al., 2023; Li, 2024). Insufficient mastery of fundamental principles undermines the quality of decisions, weakens working-capital discipline, and elevates risk of business failure ( Rehman & Mia, 2024).

The developing-country context amplifies these challenges. In Indonesia, for instance, the entrepreneurship ratio was reported at 3.47%, below the 4% threshold commonly cited for developed economies, despite a large absolute number of entrepreneurs and strong student aspirations to found businesses ( Katadata, 2023; Kemenkopukm, 2023). This structural gap suggests that building robust financial capabilities among student entrepreneurs is not merely complementary but foundational to venture viability and growth.

Theoretically, financial literacy can be framed as human capital that enhances productivity and entrepreneurial performance ( Becker, 1993). Within entrepreneurship education frameworks, integrating finance competencies through active, practice-based learning is expected to strengthen entrepreneurial intentions and action ( Fayolle & Liñán, 2014). From the lens of the Theory of Planned Behavior, stronger financial literacy increases perceived behavioral control, thereby improving the likelihood of sound entrepreneurial decision-making ( Ajzen, 1991). Although prior studies and reviews have examined financial literacy and entrepreneurship, there remains a lack of systematic synthesis focused specifically on student entrepreneurship in developing countries, and on how financial competencies are embedded within higher-education entrepreneurship curricula and interventions. Addressing this gap, the present systematic literature review (PRISMA 2020) analyzes the role of financial literacy in student entrepreneurship with an emphasis on developing-country contexts.

### Objectives

This review pursues three objectives: (1) to identify the types of financial skills addressed in research on student entrepreneurship; (2) to assess approaches and interventions used to improve students’ financial literacy; and (3) to examine the impacts of financial literacy on entrepreneurial outcomes such as intentions, decision quality, cash-flow resilience, and venture sustainability.

## Methods

### Research design

This review followed the PRISMA 2020 guideline ( Page et al., 2021), comprising four phases: identification, screening, eligibility, and inclusion.

### PRISMA procedure

**A. Identification**

Relevant literature was identified through the Scopus database due to its broad coverage of peer-reviewed journals. The search used Publish or Perish software ( Harzing, 2024). The primary keyword string was:

(“financial literacy” AND entrepreneurship AND student*) OR (“financial literacy” AND “youth entrepreneurship”)

The search covered 2014–2024 and yielded 155 records. Visualization of results using Publish or Perish is presented in Figure 2.

**B. Screening**

Records were screened by title and abstract to remove studies that were not student-focused or unrelated to entrepreneurship. Two independent reviewers conducted the screening and resolved disagreements through discussion.

**C. Eligibility**

Full-text assessment evaluated methodological clarity, topical fit, and validity of findings.

**D. Inclusion**

Studies meeting all criteria were retained for synthesis. Inclusion and exclusion criteria were defined a priori to ensure relevance and rigor ( Tod, 2019).

**Inclusion and Exclusion Criteria**

Inclusion: (i) 2014–2024; (ii) peer-reviewed empirical studies (quantitative, qualitative, or mixed methods); (iii) focus on students in an entrepreneurship context; (iv) English language. Exclusion: non-empirical articles, studies outside entrepreneurship education, or non-English sources. After screening 155 records, 36 studies were included in the final synthesis. The selection process of studies is illustrated in Figure 3. The identified records were categorized by document type as shown in Table 1.

**Figure 1.**
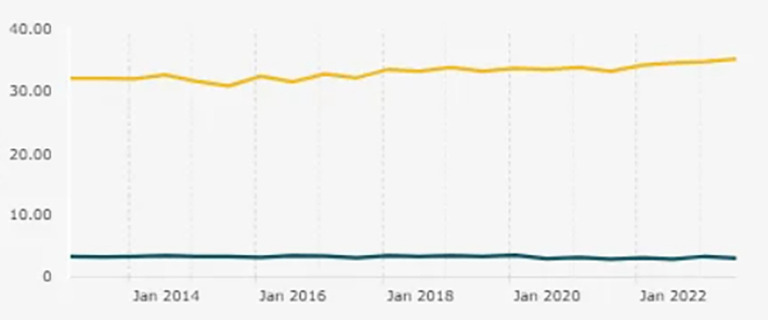
Entrepreneurship Ratio in Indonesia. Note: This figure/table has been reproduced with permission from Katadata (2023).

**Figure 2.**
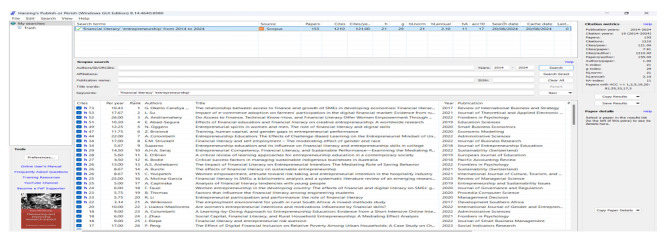
Scopus keyword search using Publish or Perish.

**Figure 3.**
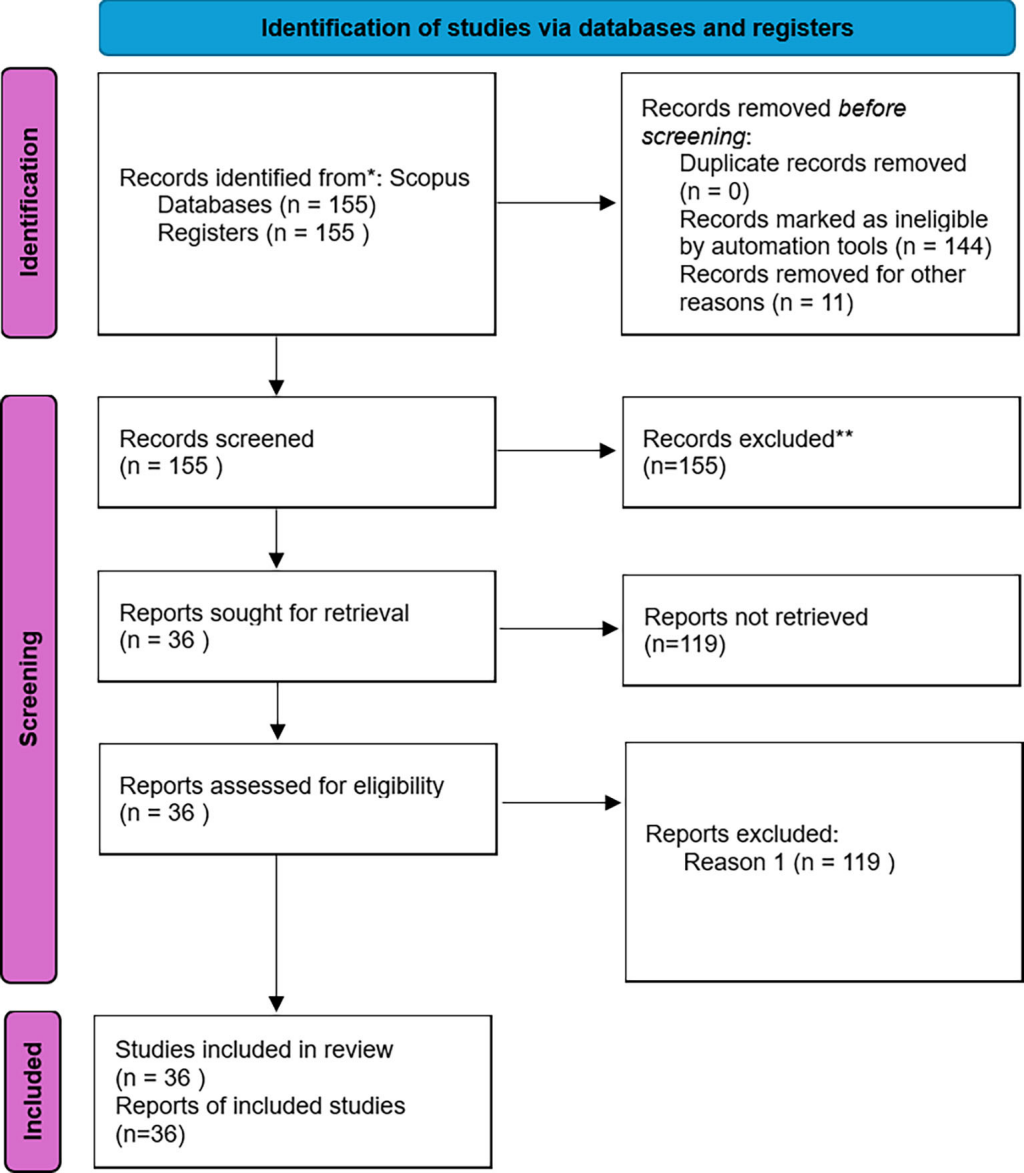
PRISMA flow diagram.

**Table 1.** Sorting documents.

Document type	Sum
Article	115
Book	1
Book Chapters	16
Conference Papers	20
Editorials	1
Reviews	3

## Results and Discussion

Findings are organized according to the three research objectives to improve clarity and interpretability. The national entrepreneurship ratio that contextualizes this issue is shown in Figure 1.

### Objective 1: Types of Financial Skills

The analyzed studies consistently addressed budgeting and cash-flow monitoring as foundational skills, followed by saving and borrowing, risk assessment and investment, capital sourcing, and basic managerial accounting. Skill gaps were most pronounced in investment analysis and capital structure among student populations in developing countries.

### Objective 2: Approaches to Improving Financial Literacy

Effective approaches included integrated finance modules within entrepreneurship curricula, case-based learning and business simulations, student venture projects, and mentoring by banks/fintech. Practice-based interventions demonstrated stronger gains in self-efficacy and decision quality compared to lecture-only formats.

### Objective 3: Impacts on Entrepreneurial Outcomes

Higher financial literacy was associated with stronger entrepreneurial intentions, improved financial decision-making, greater cash-flow resilience, and better venture sustainability. These patterns align with the Theory of Planned Behavior through enhanced perceived behavioral control. Compared with previous reviews, these findings foreground the importance of finance competencies for student founders in developing countries.

Furthermore, cross-regional evidence reveals contextual differences in how financial literacy is integrated into entrepreneurship education across developing countries. In **Asian contexts** such as Indonesia, Malaysia, and China, financial literacy programs are commonly delivered through **curriculum-integrated modules, digital business simulations, and university-based incubation initiatives** that emphasize structured, formal learning. Conversely, studies from **African developing economies**, including Ghana and Nigeria, highlight **informal mentoring systems, peer-to-peer learning, and microfinance-based entrepreneurship training** as dominant approaches. These distinctions suggest that while financial literacy universally enhances entrepreneurial outcomes, its implementation and effectiveness are influenced by each region’s educational infrastructure, economic environment, and cultural orientation. Recognizing these variations is critical for designing more context-sensitive entrepreneurship education policies and pedagogical models that promote financial capability among students globally.

A summary of the key characteristics of the reviewed studies is presented in Table 2.

**Table 2.** Synthesis of included studies (excerpt).

Author-Year	Country	Design	Sample	Financial Skills	Intervention	Outcomes	Key Findings
Li, 2024	China	Survey	350 students	Budgeting; Investment	Finance course	Entrepreneurial intention	Literacy increases readiness
Abdul Karim et al., 2023	Malaysia	Survey	492 students	Saving; Risk	—	Fraud vulnerability	Low literacy linked to vulnerability

## Conclusion

This review confirms financial literacy as core human capital for student entrepreneurship in developing countries. Critical skills include budgeting and cash-flow management, saving and borrowing, risk assessment and investment, and basic accounting. Practice-oriented curricular approaches and industry partnerships are most effective in strengthening literacy and improving entrepreneurial outcomes. Integrating financial literacy within entrepreneurship education can enhance decision quality, resilience, and venture sustainability among student founders.

## Recommendations

• Develop entrepreneurship-specific financial literacy modules and experiential finance labs.
• Build collaborations among universities, industry partners, and financial institutions to deliver mentoring and simulation-based training.
• Embed assessment of financial decision quality and cash-flow resilience into program evaluation metrics.

## Data Availability

No data are associated with this article. *Reporting guidelines* Zenodo: PRISMA Flow Diagram for Why is it important? Financial Literacy in Students in Entrepreneurship: A Systematic Literature Review [Data set]. Zenodo. https://doi.org/10.5281/zenodo.14639718 ( Romadhon & Mulyadi, 2025).

## Software availability statement

The software tools mentioned in the article are as follows:

**Publish or Perish:** This software is open-access and was used to retrieve bibliometric data for the PRISMA methodology. A hyperlink to the software has been added in the revised manuscript: https://harzing.com/resources/publish-or-perish/windows.

**Mendeley Citation Manager:** This software is a proprietary tool but is freely available for reference management purposes. To address your suggestion, we have provided an additional note in the Software Availability statement to indicate Mendeley’s availability and functionality. This has been acknowledged in the revised manuscript.

**Software availability:** The Publish or Perish software used for bibliometric analysis is freely available at https://harzing.com/resources/publish-or-perish/windows. Mendeley, a citation management tool used in this study, is freely available for download at https://www.mendeley.com.
