# Supplementary material for: Why is it important? Financial literacy in students in entrepreneurship: A systematic literature review
Source: F1000Res. 2025 Oct 31;14:138. Originally published 2025 Jan 27. [Version 3] doi: 10.12688/f1000research.160829.3 (PMC12640493; doi:10.12688/f1000research.160829.3)
Supplement: Peer review history

# Reviewer report: Reviewer response for version 3

**DOI:** 10.5256/f1000research.190540.r429288

**Published:** 2025-11-22

**Authors:** Binod Ghimire

**Affiliations:** 1 Tribhuvan University, Kirtipur, Nepal

The study offers valuable insights, supported by a strong rationale and well-formed conclusions. However, it would benefit from a clearer explanation of how each key conclusion is directly supported by specific evidence. If any contextual factors influence the findings, these should also be addressed. Additionally, the authors should elaborate on the broader implications for policymakers, educators, and curriculum designers.

Are the rationale for, and objectives of, the Systematic Review clearly stated?

Yes

Is the statistical analysis and its interpretation appropriate?

Not applicable

If this is a Living Systematic Review, is the ‘living’ method appropriate and is the search schedule clearly defined and justified? (‘Living Systematic Review’ or a variation of this term should be included in the title.)

Not applicable

Are sufficient details of the methods and analysis provided to allow replication by others?

Yes

Are the conclusions drawn adequately supported by the results presented in the review?

Yes

Reviewer Expertise:

Behaviour Finance, Organizational Behaviour, Stock Market

I confirm that I have read this submission and believe that I have an appropriate level of expertise to confirm that it is of an acceptable scientific standard.

# Reviewer report: Reviewer response for version 3

**DOI:** 10.5256/f1000research.190540.r421963

**Published:** 2025-11-07

**Authors:** Nurul Hidayana Mohd Noor

**Affiliations:** 1 Faculty of Administrative Science and Policy Studies, Universiti Teknologi MARA, Seremban, Negeri Sembilan, Malaysia

All revisions have been made accordingly in response to the reviewers’ comments. The manuscript has been thoroughly amended and improved based on the feedback provided.

Are the rationale for, and objectives of, the Systematic Review clearly stated?

Partly

Is the statistical analysis and its interpretation appropriate?

Partly

If this is a Living Systematic Review, is the ‘living’ method appropriate and is the search schedule clearly defined and justified? (‘Living Systematic Review’ or a variation of this term should be included in the title.)

Partly

Are sufficient details of the methods and analysis provided to allow replication by others?

Partly

Are the conclusions drawn adequately supported by the results presented in the review?

Partly

Reviewer Expertise:

organizational and nonprofit management, entreprenuership, public policy and governance, and environmental management

I confirm that I have read this submission and believe that I have an appropriate level of expertise to confirm that it is of an acceptable scientific standard.

# Reviewer report: Reviewer response for version 2

**DOI:** 10.5256/f1000research.188832.r421962

**Published:** 2025-10-29

**Authors:** Muhammad Hasan

**Affiliations:** 1 Universitas Negeri Makassar, Makassar, Indonesia

This revised version demonstrates a notable improvement in both structure and substance compared to the previous submission. The authors have clearly responded to prior reviewer feedback by enhancing the clarity of the title, strengthening the theoretical framework, and situating the study more firmly within the context of developing countries—especially Indonesia. The integration of Human Capital Theory and the Theory of Planned Behavior add conceptual rigor that was previously missing, while the expanded introduction now offers a stronger justification for the study’s relevance and novelty.

 The methodology section shows significant progress. The description of the PRISMA 2020 process, inclusion and exclusion criteria, and the visualization of the search procedure through Publish or Perish now ensure methodological transparency and replicability. Although reliance on Scopus remains a limitation, the acknowledgment of this constraint and the suggestion for future inclusion of WoS and ERIC are appropriate and strengthen the paper’s integrity.

 The “Findings and Discussion” section is now better structured around the stated objectives, making it easier to follow. Thematic organization by types of financial skills, pedagogical approaches, and impacts on entrepreneurial outcomes enhances interpretability and aligns with PRISMA’s systematic synthesis standards. Compared with the earlier version, the narrative demonstrates deeper engagement with the literature, and the inclusion of developing-country insights adds value to the global discussion on financial literacy and student entrepreneurship.

 The conclusion and recommendations are more aligned with the findings. They now clearly articulate theoretical, practical, and policy implications, particularly in terms of integrating financial literacy into entrepreneurship education and fostering collaboration among universities, financial institutions, and policymakers. These revisions meaningfully elevate the scholarly and applied contribution of the manuscript.

 Overall, the authors have addressed most of the critical weaknesses highlighted in the first review, including issues of novelty, organization, methodological clarity, and theoretical grounding. The paper now meets an acceptable scientific standard for indexing, with only minor polishing needed in language consistency and deeper reflection on cross-regional comparisons in the discussion section.

Are the rationale for, and objectives of, the Systematic Review clearly stated?

Yes

Is the statistical analysis and its interpretation appropriate?

Not applicable

If this is a Living Systematic Review, is the ‘living’ method appropriate and is the search schedule clearly defined and justified? (‘Living Systematic Review’ or a variation of this term should be included in the title.)

Not applicable

Are sufficient details of the methods and analysis provided to allow replication by others?

No

Are the conclusions drawn adequately supported by the results presented in the review?

No

Reviewer Expertise:

Entrepreneurship education, economics literacy, economics education, knowldge transfer.

I confirm that I have read this submission and believe that I have an appropriate level of expertise to confirm that it is of an acceptable scientific standard, however I have significant reservations, as outlined above.

# Response

**Published:** 2025-10-29

**Authors:** Dwi Romadhon

**Affiliations:** FPEB, Universitas Pendidikan Indonesia, Bandung, West Java, Indonesia

We sincerely thank the reviewer for the constructive and encouraging feedback. We appreciate the recognition of the major improvements in this revised version and have carefully addressed the remaining suggestions as follows:

1. **Language Consistency**
  The entire manuscript has been re-edited for uniformity of academic style and grammatical accuracy. Terminologies such as *financial literacy*, *entrepreneurial outcomes*, and *student entrepreneurship* have been standardized. We also improved transitions between sections and verified citation formats to ensure stylistic coherence throughout the text.
2. **Cross-Regional Reflection**
  Following the reviewer’s valuable suggestion, the *Discussion* section has been expanded to include a comparative analysis across developing-country contexts. The new paragraph emphasizes differences in the design and implementation of financial-literacy initiatives between **Asian settings (Indonesia, Malaysia, China)** and **African contexts (Ghana, Nigeria)**.
  Specifically, Asian programs tend to focus on **curriculum-integrated finance modules and digital business simulations**, while African studies highlight **community-based mentoring, informal entrepreneurship training, and micro-finance exposure**. This addition provides broader interpretive depth and strengthens the article’s contribution to understanding how socio-economic and educational structures influence financial-literacy outcomes globally.
3. **Minor Editorial Revisions**
  Figures, tables, and in-text references have been checked for consistency, and formatting was adjusted to meet journal standards. These refinements improve clarity and readability.

We believe these revisions satisfactorily address the reviewer’s final concerns. We greatly appreciate the thoughtful review and constructive guidance, which have enhanced both the scholarly depth and overall presentation of the manuscript.

# Reviewer report: Reviewer response for version 1

**DOI:** 10.5256/f1000research.176781.r370750

**Published:** 2025-04-02

**Authors:** Muhammad Hasan

**Affiliations:** 1 Universitas Negeri Makassar, Makassar, Indonesia

Dear Author,

 Congratulations on successfully completing this manuscript. I have read it with great interest. There are several problems that need to be addressed in this manuscript.

 First, in the introduction. You have not specifically described the novelty of this finding. There have been many literature review themes like this. However, you have not explained what distinguishes the findings of this study from previous studies. My suggestion is to explore it with the perspective of entrepreneurship education in developing countries. In addition, the theoretical perspective you use has not been described specifically.

 Second, in the results section. This section is very weak. There is no information found by the reader, especially the latest information. This section is unable to reduce the entire literature that has been reviewed.

 Third, the conclusion section. Not in line with the research results. In addition, the recommendations and/or theoretical and practical implications are unclear.

Are the rationale for, and objectives of, the Systematic Review clearly stated?

Yes

Is the statistical analysis and its interpretation appropriate?

Not applicable

If this is a Living Systematic Review, is the ‘living’ method appropriate and is the search schedule clearly defined and justified? (‘Living Systematic Review’ or a variation of this term should be included in the title.)

Not applicable

Are sufficient details of the methods and analysis provided to allow replication by others?

No

Are the conclusions drawn adequately supported by the results presented in the review?

No

Reviewer Expertise:

Entrepreneurship education, economics literacy, economics education, knowldge transfer.

I confirm that I have read this submission and believe that I have an appropriate level of expertise to state that I do not consider it to be of an acceptable scientific standard, for reasons outlined above.

# Response

**Published:** 2025-04-02

**Authors:** Dwi Romadhon

**Affiliations:** FPEB, Universitas Pendidikan Indonesia, Bandung, West Java, Indonesia

Dear Reviewer,

 Thank you for your valuable and constructive feedback on our manuscript titled "Why is it important? Financial literacy in students in entrepreneurship: A systematic literature review." We appreciate your time and effort in reviewing the manuscript. Below are our responses to your comments and the revisions we have made:

 1. Novelty and Theoretical Framework in the Introduction

 We acknowledge the concern regarding the lack of a clear explanation of the novelty of our study. To address this, we have revised the introduction to explicitly highlight the unique contribution of our research. Specifically, we have emphasized how our study explores entrepreneurship education in developing countries, a perspective that has not been adequately addressed in previous literature. Additionally, we have provided a more detailed description of the theoretical framework, focusing on the integration of financial literacy within entrepreneurship education and its significance in the context of developing countries.

 2. Weak Results Section

 We agree that the results section lacked sufficient clarity and recent insights. In response, we have strengthened this section by incorporating the most current literature and synthesizing the findings in a more comprehensive manner. We have clearly presented key trends, gaps, and challenges related to financial literacy among students, particularly in developing countries, and how these factors impact entrepreneurship education.

 3. Conclusion and Implications

 We understand that the conclusion did not align well with the research findings. We have revised the conclusion to ensure it directly reflects the results of the systematic review. Furthermore, we have expanded the recommendations to include both theoretical and practical implications for enhancing financial literacy in entrepreneurship education. This includes suggestions for policymakers, educators, and curriculum developers in developing countries.

 4. Methodology and Replicability

 We recognize that the methodology section required more detail. In response, we have elaborated on the systematic review process, providing clear information on the selection criteria, data sources, and analysis methods used in our study. This ensures that the methodology is transparent and allows for replication by other researchers in the field.

 We hope that these revisions address your concerns and improve the clarity and quality of the manuscript. Thank you once again for your thoughtful feedback. We look forward to your continued guidance.

 Sincerely,

 Dwi Nanda Akhmad Romadhon & Hari Mulyadi

 Authors

# Reviewer report: Reviewer response for version 1

**DOI:** 10.5256/f1000research.176781.r370755

**Published:** 2025-03-25

**Authors:** Nurul Hidayana Mohd Noor

**Affiliations:** 1 Faculty of Administrative Science and Policy Studies, Universiti Teknologi MARA, Seremban, Negeri Sembilan, Malaysia

“Why is it important? Financial literacy in students in entrepreneurship: A systematic literature review” – **the title** is confusing. Does it include students who are involved in entrepreneurship?

 The methodology is not clearly explained in the abstract.

 In the **introduction**, the authors need to deliberate on the impact of entrepreneurship on the country, economy, and society. Explain also how entrepreneurship is significant in the Indonesian context. Then, down to a discussion on youth entrepreneurship. Explain the growth, highlight the challenges and issues, and then focus on the importance of financial management.

 Financial skills – define and elaborate on the skills and what is still lacking among youth in Indonesia.

 Please highlight the research gaps and discuss the significance of the study.

 The literature review section needs to be included in the manuscript.

**Methodology**

 Why did the researchers include the SCOPUS journal only? How about WOS?

 Scopus keyword search needs to be included in a written form.

 Figure 3 is not a result. You may organize it as “Findings and Discussion.”

 The findings section is poor, with a lack of in-depth discussion. Please thoroughly discuss the findings.

 The study should have clear research objectives to help the researchers to organize the findings.

 For example:

1. To assess types of financial skills

Findings and Discussion

  

1. To assess the approach to improving financial literacy

Findings and Discussion

 Thus, you can structure the discussion section based on the PRISMA findings

 The current structure is disorganized and difficult to understand.

 Recommendations – needs improvement as not explained properly.

Are the rationale for, and objectives of, the Systematic Review clearly stated?

Partly

Is the statistical analysis and its interpretation appropriate?

Partly

If this is a Living Systematic Review, is the ‘living’ method appropriate and is the search schedule clearly defined and justified? (‘Living Systematic Review’ or a variation of this term should be included in the title.)

Partly

Are sufficient details of the methods and analysis provided to allow replication by others?

Partly

Are the conclusions drawn adequately supported by the results presented in the review?

Partly

Reviewer Expertise:

Entrepreneurship. social and youth studies, management, and environmental studies

I confirm that I have read this submission and believe that I have an appropriate level of expertise to confirm that it is of an acceptable scientific standard, however I have significant reservations, as outlined above.

# Response

**Published:** 2025-03-25

**Authors:** Dwi Romadhon

**Affiliations:** FPEB, Universitas Pendidikan Indonesia, Bandung, West Java, Indonesia

1. We appreciate the reviewer’s comment regarding the title of the article. The primary objective of this article is to provide a systematic review of financial literacy among students who are either engaged in or interested in pursuing entrepreneurship. The title was designed to attract readers by highlighting the importance of financial literacy for student entrepreneurs.

 2. We agree and have revised the abstract to clearly state the use of the PRISMA methodology, inclusion criteria, and number of studies selected for review. The revised version includes:

 “This study systematically reviews 36 peer-reviewed articles from Scopus indexed journals using PRISMA guidelines (Preferred Reporting Items for Systematic Reviews and Meta-Analyses) from 2014 to 2024...”

 3. We have restructured the introduction by:

- Elaborating on entrepreneurship's macroeconomic contributions.
- Including data from Indonesia’s Ministry of Cooperatives and SMEs.
- Highlighting the growing aspirations among youth for entrepreneurship, the persistent low entrepreneurship ratio, and financial challenges as a key barrier

4. In the introduction and discussion sections, we have included a comprehensive definition of financial skills, including budgeting, debt management, savings planning, investment decision-making, and risk analysis. We also emphasize empirical findings about gaps in Indonesian youth’s understanding, especially related to debt and long-term financial planning.

 5. We added a dedicated paragraph in the final section of the introduction, explaining:

- The lack of integration of financial literacy in entrepreneurship education curricula.
- Inadequate empirical exploration of financial behavior among student entrepreneurs.
- The need for a framework that connects financial skills to entrepreneurial success among youth in Indonesia.

6. We have now added a distinct section titled **“Literature Review”**, which summarizes relevant theories, prior findings, and trends over the last 10 years concerning student entrepreneurship and financial literacy.

 7. We have now clarified in the methodology section that:

 “Scopus was selected due to its comprehensive coverage of peer-reviewed journals and practical access limitations. However, we acknowledge that future reviews should include WoS and ERIC to broaden the perspective.”

 8. We have now written explicitly in the methodology section:

 “The keywords used in Scopus via Publish or Perish were: (‘financial literacy’) AND (‘entrepreneurship’) AND (‘students’ OR ‘youth’). Search filters included publication year (2014–2024), English language, and peer-reviewed sources.”

 9. Figure 3 (PRISMA diagram) has been moved to the Method section, and we have renamed the third section to **“Findings and Discussion”** to reflect the synthesis of reviewed literature and align with PRISMA flow.

 10.  We agree. The discussion section has now been expanded with:

- Thematic analysis of the 36 selected studies (e.g., types of financial skills emphasized, teaching approaches used, regional contexts, outcomes).
- A synthesis comparing financial education practices across countries.
- Implications for Indonesian higher education and policy.

11. We now include specific objectives at the end of the introduction:

1. To identify the types of financial skills essential for student entrepreneurs.
2. To analyze current approaches to improving financial literacy among student entrepreneurs.
3. To develop implications for integrating financial literacy in entrepreneurship education.

These objectives are used to structure the **Findings and Discussion** section accordingly.

 12. The **Recommendations** section has been expanded to include:

- Proposals for module development and integration into curricula.
- Collaboration models between universities, banks, and entrepreneurs.
- Policy recommendations for higher education authorities in Indonesia.

13. Summary comments: We have thoroughly revised the manuscript to address all these concerns:

- Objectives clearly stated.
- PRISMA methodology explained with flow diagram and inclusion criteria.
- No statistical meta-analysis was conducted, but thematic synthesis was applied and described.
- All conclusions are now tightly aligned with the synthesized literature findings.
